# A Systematic Review Exploring the Anticancer Activity and Mechanisms of Glucomannan

**DOI:** 10.3389/fphar.2019.00930

**Published:** 2019-08-23

**Authors:** Jun-yi Li, Fei Sun, Hai-feng Zhou, Jia Yang, Cong Huang, Heng Fan

**Affiliations:** ^1^Department of Integrated Traditional Chinese and Western Medicine, Union Hospital, Tongji Medical College, Huazhong University of Science and Technology, Wuhan, China; ^2^The Center for Biomedical Research, Tongji Hospital, Tongji Medical College, Huazhong University of Science and Technology, Wuhan, China; ^3^Institute of Integrated Traditional Chinese and Western Medicine, Tongji Hospital, Tongji Medical College, Huazhong University of Science and Technology, Wuhan, China

**Keywords:** glucomannan, polysaccharide, apoptosis, drug carrier, anti-tumor effect

## Abstract

Glucomannan, long recognized as the active ingredient of the traditional Chinese medicinal herb Konjac glucomannan, is a naturally occurring polysaccharide existing in certain plant species and fungi. Due to its special property to also serve as a dietary supplement, glucomannan has been widely applied in clinic to lower body weight and circulation cholesterol level and to treat constipation, diabetes, and arterial sclerosis. Besides the regulatory role engaged with gastroenterological and metabolic syndrome, recently, its therapeutic effect and the underlying mechanisms in treating cancerous diseases have been appreciated by mounting researches. The present review aims to emphasize the multifaceted aspects of how glucomannan exerts its anti-tumor function.

## Introduction

Currently, tumors are increasingly becoming a global health issue. According to an epidemiological survey, cancer already surpasses cardiovascular and cerebrovascular accidents, and ranks as the top life-threatening disease worldwide. A status report on the global burden of cancer using GLOBOCAN estimated 18.1 million new cancer cases and 9.6 million cancer deaths in 2018 year alone (Bray et al., 2018). The overall cancer (containing more than 18 types of cancer) 5-year survival rates in China, Japan, and the United States were 36.0%, 57.4%, and 64.0% (Allemani et al., 2018), which suggests a significant variation among different countries.

The reasons that make cancer so tough to deal with are complicated. Basically speaking, dysfunctional cell cycle regulation with an excessive proliferation capacity and undifferentiated immature phenotype are the hallmarks of cancer pathology. Besides, tumor cells show intrinsic aberrance of multiple cellular processes. High expression of pro-survival genes like *BCL-2* and *MCL1* render them apoptosis resistant (Sawai et al., 2018), and metabolic pathways are rewired to make tumor cells better adapt to the changing environment. Tumor cells are experts in nutrient utilization; they adopt distinct metabolic programs, ranging from glycolysis, fatty acid oxidation, to amino acid metabolism under various circumstances. Accompanied by the elevated metabolic activity required for uncontrolled growth, the production of reactive oxygen species (ROS) is substantially increased, which further causes genomic instability and accumulation of genetic mutation (Sabharwal and Schumacker, 2014). Interestingly, autophagy seems to play a dual role in tumor development. During the early stage, autophagy inhibits tumor formation through alleviating cellular stress; however, this mechanism is then hijacked by later-stage tumor cells to facilitate their growth under extreme conditions. To make things awkward, tumors could also have active impact on the microenvironment where they live. VEGF-C from tumor cells promotes blood and lymphatic vessel neogenesis, which is in favor of tumor cells’ development and metastasis (Liu et al., 2019). Meantime, immune cells are educated into a tolerant or anergic state, thus leading to tumor immune evasion.

Researches on cancer treatment are focused on two aspects: one is to discover new anti-tumor therapy, while another is to establish an efficient drug-delivery system. With the advance of medical science, we now possess huge artillery of weapons against tumor. Conventional protocols including surgical removal, radio-therapy, and chemotherapy form the basis of clinical therapies. Strikingly, immunological approaches are emerging as a strong supplement. The deployment of cell-based CAR-T (Abramson et al., 2017) or DC-CIK strategy (Zhang et al., 2018) and usage of checkpoint inhibitors (anti-PD-1, anti-CTLA4, etc.) (Wei et al., 2017) as well as tumor vaccines (Kaiser, 2017) bring about great benefits for certain populations of patients. To further improve the selectivity, biomarker-discovery-based monoclonal antibody development and finely designed nanoparticle carriers are applied. Nonetheless, these measures are not enough to win the battle against tumors. The side effect, off-target effect, and loss of effect are prevalent phenomena among present therapeutic protocols. Thus, it is of importance to pursue additional methods and bioactive anti-tumor compounds.

Glucomannan is a family of polysaccharide widely existing in higher plants and microorganisms. The backbone structure is β-(1→4) glycoside bond linked D-mannose and D-glucose in a ratio of 1.6:1, while it is only lightly branched through β-(1→6) glucosyl moieties (Wu et al., 2011a). The molecular weight ranges from 200,000 to 2,000,000 Daltons, which varies with origin, method of processing, and storage time. In general, glucomannan has favorable characteristics of water solubility and extremely low toxicity (Hassan et al., 2014; Luan et al., 2017), which make it a fantastic bio-compatible compound. Actually, Konjac glucomannan, the well-known member of the glucomannan family, was first documented as a traditional Chinese herb about 2000 years ago (CP C, 2010). Since then, it has been used to treat conditions such as asthma, cough, hernia, breast pain, burns, and hematological and skin diseases (CP C, 2010). Nowadays, it mainly serves either as a dietary supplement aiding in weight loss, diabetes, arterial sclerosis, and constipation, or as an emulsifier and thickener in food processing (Chen et al., 2017a; Wu et al., 2018), which corroborates the old theory of “homology of medicine and food.” In fact, many natural polysaccharides that show potent anti-tumor activity (Kim et al., 2011; Xiao et al., 2017; Zhang, 2017; Deng et al., 2018) derive from edible herbs, such as *Lentinus*, *Cordyceps*, *Ganoderma lucidum*, and *Hericium*, which themselves are also common cuisine ingredients. Recently, glucomannan extracted from *Amorphophallus konjac* (Chen et al., 2017a), *Bletilla striata* (Zhan et al., 2014), *Lentinus edodes* (Fujii et al., 1978), *Aloe vera* (Sampedro et al., 2004; Im et al., 2005; Liu et al., 2006; Im et al., 2016; Quezada et al., 2017), and *Candida utilis* (Kumano et al., 1985) were demonstrated to have similar effect. Moreover, according to the studies reviewed in the present research, glucomannan, mainly from these species, not only is an anti-cancer drug by itself but also works as a targeted carrier that is compatible with various bioactive compounds.

## Glucomannan Directly Interferes With Intrinsic Tumor Cells Biological Processes

### Glucomannan Impairs Tumor Cells’ Survival and Metastasis

**Figure 1:**
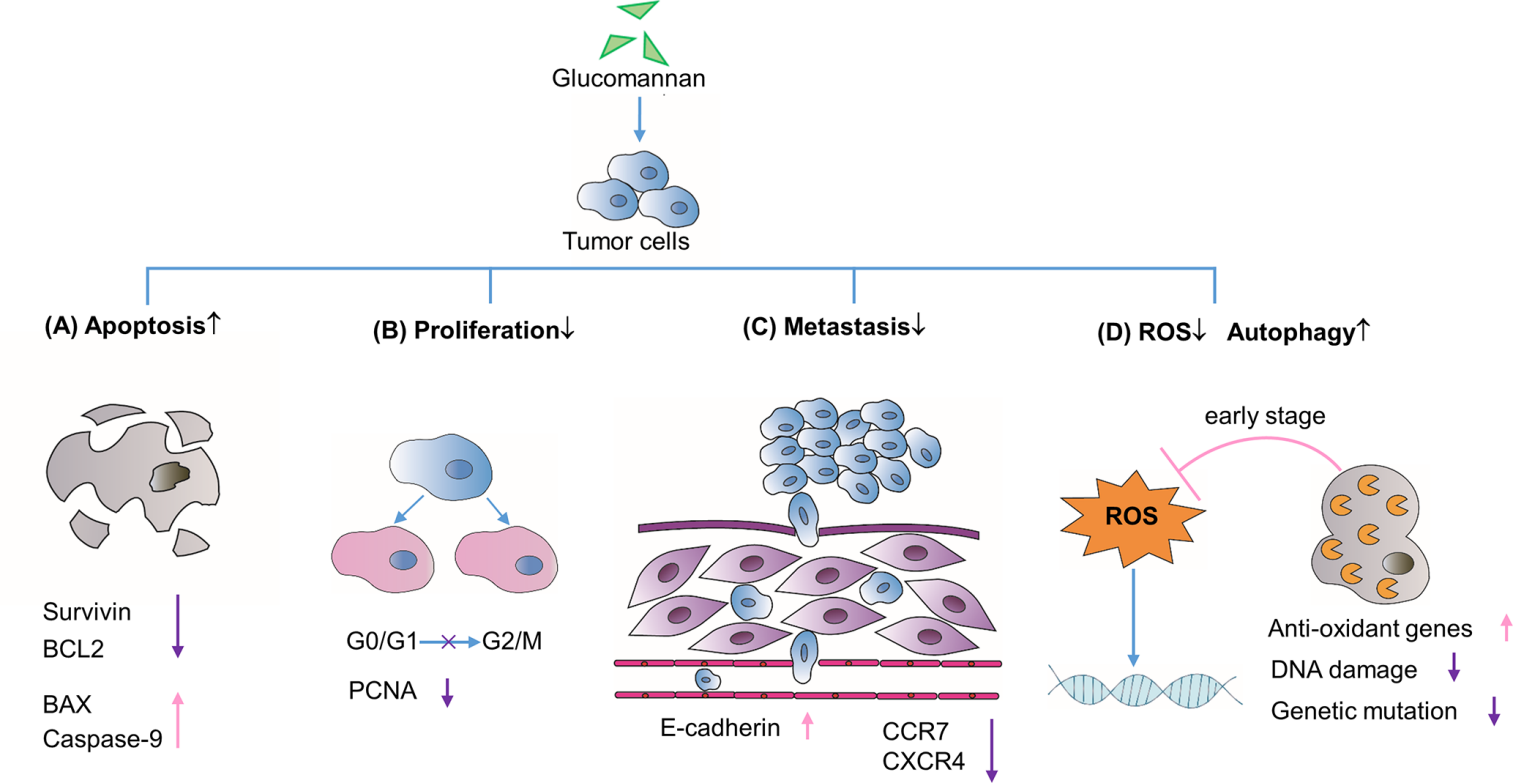
Direct effects on tumor cell biological processes. **(A)** Glucomannan promotes tumor cell apoptosis through the up-regulation of pro-apoptotic proteins BAX and Caspase-9 and the down-regulation of anti-apoptotic genes like Survivin and BCL2. **(B)** Glucomannan inhibits tumor cell proliferation through the blockade of G0/G1 to G2/M phase transition and decreases the expression of PCNA. **(C)** Glucomannan hinders tumor cell metastasis by decreasing CCR7 and CXCR4 expression and increasing E-cadherin level. **(D)** Glucomannan promotes autophagy and reduces ROS production in tumor cells, thus leading to the alleviation of DNA damage and subsequent mutation events.

Given that tumor cells are apoptosis resistant, drugs aimed to promote apoptosis are widely used in cancer therapy (Hassan et al., 2014). Initially, studies unveiled the therapeutic effect of extract from *A. konjac* (AKe) on various cancers, such as colon carcinoma (Ansil et al., 2013; Ansil et al., 2014a), gastric cancer (Chen et al., 2017a), hepatoma (Ansil et al., 2014b), and breast cancer (Wu et al., 2018; Wu et al., 2019). Chen et al. found that AKe could inhibit the growth of *in vitro* cultured gastric cancer cell lines SGC-7901 and AGS (Chen et al., 2017a). On the molecular level, the expression of inhibitors of apoptosis protein (IAP) family member survivin and the pro-survival gene *BCL2* decreased, while the pro-apoptosis protein BAX and caspase-9 increased (Chen et al., 2017a) (**Figure 1**). AKe also displayed similar therapeutic effect on human liver cancer cell line and the human triple-negative breast cancer (TNBC) cells (Ansil et al., 2014b; Wu et al., 2018). By blocking the transition from G0/G1 to G2/M phase and decreasing the expression of the proliferative marker proliferative cell nuclear antigen (PCNA), AKe promotes cell cycle arrest and inhibits cell division (Ansil et al., 2013; Chen et al., 2017a) (**Figure 1**). The elevated activity of tumor cell apoptosis is always accompanied by decreased cell proliferation, which stems from the shared down-regulated PI3K/AKT signaling pathway (Frisch and Screaton, 2001; Guanen et al., 2018). Since Konjac glucomannan was identified as the major bioactive component of the *A. konjac* (Chen et al., 2017a), not surprisingly, Sawai S. and his colleagues found that Konjac-glucomannan-treated HepG2 hepatic carcinoma cells displayed a significant reduction of growth, which resembles the phenotype of AKe-treated tumor cells (Sawai et al., 2018).

Indeed, the PI3K/AKT/mTOR pathway is frequently hyper-activated in tumor cells and also plays an important role in cancer metastasis (Guanen et al., 2018). AKe was found to inhibit PI3K signaling pathway and thwart MDA-MB231 breast cancer cell metastasis to lung in a xenograft model (Wu et al., 2018). The chemokine receptors and adhesion molecules are strongly linked to tumor recurrence and metastasis. AKe was reported to significantly increase E-cadherin expression and reduce CCR7 and CXCR4 level, which are required for tumor migration (Wu et al., 2018) (**Figure 1**). Moreover, the glucomannan extracted from the *C. utilis* preferentially inhibited the Lewis lung carcinoma (3LL) pulmonary metastases in a time-dependent manner, wherein an optimal dosage of glucomannan on early stage exhibits the optimal suppressive effect (Kumano et al., 1985).

Collectively, these data outline the intrinsic impacts of glucomannan on tumor cell apoptosis, proliferation, and metastatic activity, which are the essential basis for its anti-tumor function. To a large extent, such effects are attributed to glucomannan-mediated inhibition of the PI3K/AKT signaling pathway. As a result, increased ratio of pro-apoptotic to anti-apoptotic gene expression undermines the immortality of tumor cells, while the reduced level of CCR7 and CXCR4 compromises their migratory capability.

### Glucomannan Enhances Anti-Oxidative Activity and Induces Autophagy in Tumor Cells

Another glucomannan-mediated anti-cancer effect could be ascribed to its role as an antioxidant inducer (Miadoková et al., 2006; Ansil et al., 2013; Ansil et al., 2014b; Wu et al., 2014). An earlier study confirmed that glucomannan isolated from *C. utilis* exerts anti-mutagenic function in a mouse model of cyclophosphamide-induced mutagenicity, which may result from the decreased ROS level (Chorvatovicova et al., 1999). Miadoková et al. further concluded that glucomannan from *C. utilis* cell wall exerts the anti-oxidative activity through iron chelation and scavenging of hydroxyl radicals in mouse leukemia cell (Miadoková et al., 2006). In line with the above observation, glucomannan or AKe was demonstrated to enhance the expression of antioxidant enzymes in 1,2-dimethylhydrazine (DMH)-induced colon carcinogenesis and *N*-nitrosodiethylamine-induced hepatocellular carcinoma in rats (Ansil et al., 2013; Ansil et al., 2014c). Besides, Konjac glucomannan was able to ameliorate AOM-induced genotoxicity *via* alleviating the DNA damage process initiated by accumulated ROS (Wu et al., 2014) (**Figure 1**).

Alternatively, in the gastric cancer cell lines SGC-7901 and AGS, investigators found that AKe treatment markedly increased the expression of LC3-II, which is a marker of the mature autophagosome and indicates high level of autophagy (Chen et al., 2017a). Increased cell autophagy plays a protective role in early tumor development, with the possible mechanisms of autophagy-related stress elimination and even autophagy-induced cell death. Nassour et al. found that autophagy is an important cellular protective process to induce cell death during replicative crisis and impaired autophagy is required for initiation of tumorigenesis (Nassour et al., 2019). Though the AKT/mTOR pathway may be related to the activation of the autophagic process (Chen et al., 2014), there is no definite evidence showing the existence of the glucomannan-AKT/mTOR-autophagy axis in the tumor cells.

## Glucomannan Indirectly Affects Tumor Development

### Glucomannan Promotes Extrinsic Environment Disfavoring Tumorigenesis

**Figure 2:**
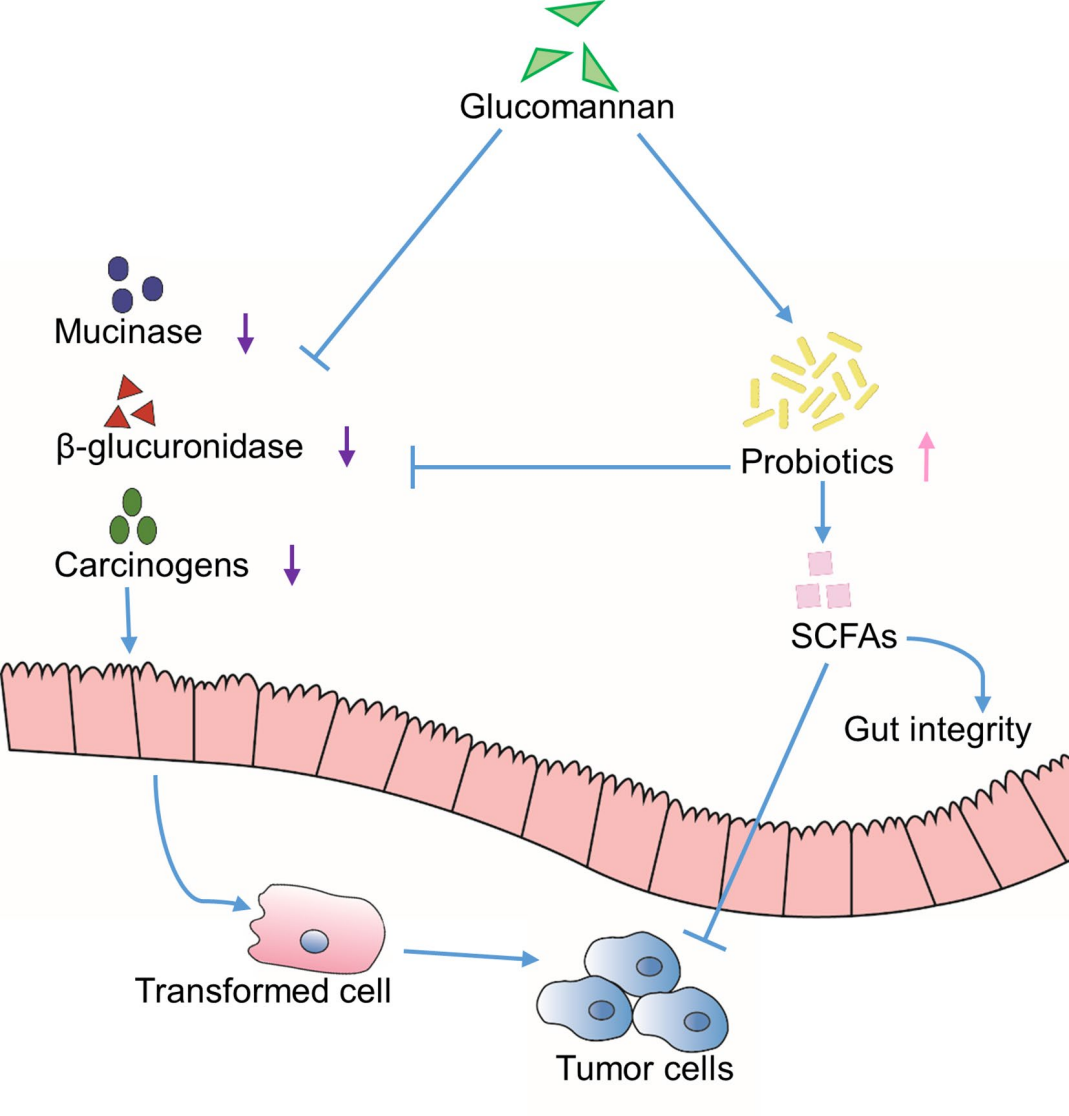
Indirect effects through extrinsic environment. Inhibition of the activity of mucinase and β-glucuronidase is achieved through glucomannan or glucomannan-induced colonization of probiotics, which results in reduced carcinogens and increased SCFAs content in the gut lumen. Altogether, the tumor development is suppressed.

Glucomannan displays a beneficial effect for constipation and promotes intestinal peristalsis. As a dietary water-absorbing fiber, it has also been demonstrated to reduce the production of carcinogens from the gut (Mizutani and Mitsuoka, 1982; Wu and Chen, 2011b; Wu et al., 2011a; Chong, 2013). Due to its hydrolytic ability of turning substances into carcinogens, β-glucuronidase is identified as a lysosomal enzyme involved in the process of tumor development and metastasis (Klinder et al., 2008). Another protein peptide hydrolase, mucinase, is able to hydrolyze the mucin layer of the gut lumen and make the enterocytes to be in direct contact with colon carcinogens (Miller and Hoskins, 1981). According to Dr. Wu’s research, the incorporation of Konjac glucomannan in high-fat fiber-free diet in rats decreased the activity of β-glucuronidase and mucinase by 71% and 68%, respectively (Wu and Chen, 2011b) (**Figure 2**).

Studies proved that bile acid is in close relationship with the carcinogenesis process (Mizutani and Mitsuoka, 1982). It could promote gastrointestinal (GI) inflammation (Jia et al., 2018) and change the composition of normal gut microbiota, by means of which bile acid becomes a risk factor during tumor formation (Jia et al., 2018). Administration of Konjac glucomannan was found to increase the fecal output of total bile acids in rats (Ikegami et al., 1984; Wu and Chen, 2011b). What’s more, Konjac glucomannan significantly decreases secondary bile acid level in human subjects (Wu et al., 2011a). Therefore, the reduction of bile acids would be another functional aspect of glucomannan supplementation.

On the other hand, the probiotics, mainly *lactobacilli* and *bifidobacteria*, are indicated for their anti-colorectal cancer function through binding to the mutagens, inhibiting the activity of β-glucuronidase, and reducing the level of carcinogenic secondary bile acid (Wu et al., 2011a; Chong, 2013). In various studies, glucomannan is shown to enhance the content of probiotics and its metabolic products, short-chain fatty acids (SCFAs), in feces (Mizutani and Mitsuoka, 1982; Ikegami et al., 1984; Yeh et al., 2007; Wu and Chen, 2011b; Wu et al., 2011a; Chong, 2013; Wu et al., 2014; Quezada et al., 2017). SCFAs promote the functionality of normal intestinal epithelium and the gut integrity (Chong, 2013); also, they stimulate cell differentiation program and induce apoptosis of transformed cells (Kumano et al., 1985; Yeh et al., 2007; Chong, 2013). Taken together, these results suggest that glucomannan could indirectly promote an environment that is unfavorable for cancer development, at least in the gastroenterological system (**Figure 2**).

### Glucomannan Serves as a Targeted Carrier for Anti-Cancer Drug Delivery

A specific drug delivery system is necessary to enhance the efficacy of existing chemotherapeutic drugs and to reduce their side effect. Glucomannan exhibits very low toxicity, modifiability, and the absence of immunogenicity (Zhan et al., 2014). These outstanding characteristics make it an ideal drug carrier.

**Figure 3:**
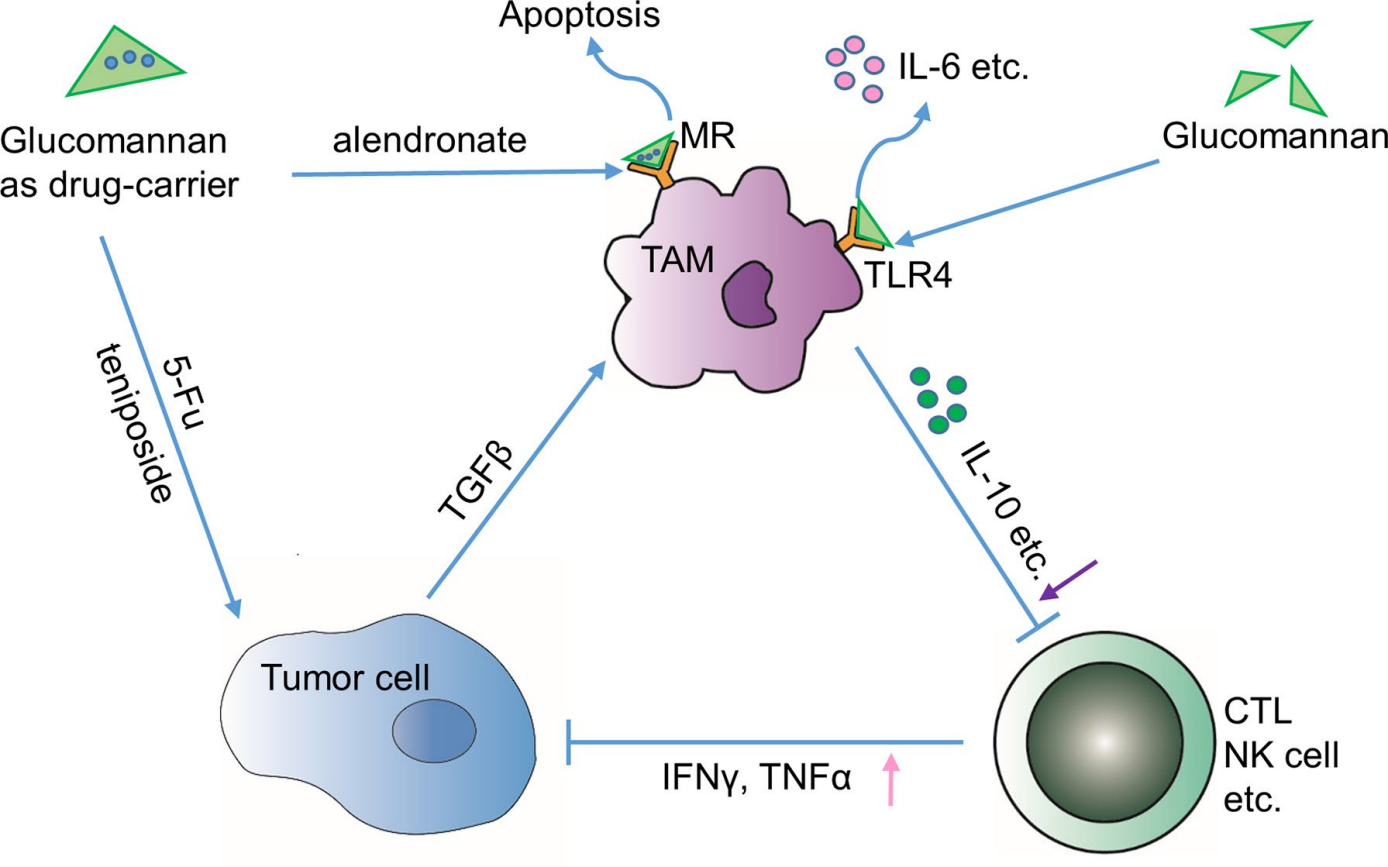
Indirect effects through tumor immune microenvironment. Glucomannan can serve as a drug carrier of anti-tumor drugs such as 5-FU and teniposide towards tumor cells or deliver apoptosis-inducing drug alendronate to TAMs through binding to mannose receptor (MR). Alternatively, glucomannan can directly bind to TLR4 receptor expressed by TAMs and leads to the secretion of IL-6, etc. In brief, administration of glucomannan reshapes the tumor immune microenvironment by increasing anti-tumor cytokines TNFα and IFN-γ and decreasing tolerance-related cytokines like IL-10.

Amphiphilic aliphatic amines grafted Konjac glucomannan micelle (Konjac glucomannan-g-AHs), a modified Konjac glucomannan, is added with a pH-sensitive Schiff’s base. With improved stability, solubility, and cytotoxicity, the Konjac-glucomannan-based micelles are able to protect the curcumin from degradation and deliver it into the tumor site through endocytosis-mediated transmembrane transport (Luan et al., 2017). Furthermore, glucomannan synergizes with other bioactive compounds to exert anti-tumor function. Combined with teniposide, they display enhanced cytotoxic and cytostatic effect on mouse leukemia cells (Miadoková et al., 2006) (**Figure 3**). In addition, the Konjac glucomannan/sodium alginate/graphene oxide (Konjac glucomannan/SA/GO) complex could effectively control the release of anti-cancer drug 5-FU in a local tumor site, thus reducing the systematic side effect (Wang et al., 2014; Yuan et al., 2019). Liu et al. designed a novel glucomannan-containing, aromatic azo agent bis(methacryloyl-amino)-azobenzene cross-linked hydrogel, which can release peptide drugs at the colon site in a pH-dependent or enzymatic degradation mode (Liu et al., 2004). Chitosan (CS)-coated microsphere, generated on the basis of oxidized Konjac glucomannan, makes another promising intestine-specific drug delivery approach for the treatment of bowel diseases, according to Shi’s study (Shi et al., 2017). In brief, glucomannan is compatible with many other bioactive compounds and the conjugated macromolecular complexes could be easily taken by tumor cells, thus improving the therapeutic efficacy of existing anti-cancer chemicals.

### The Effect of Glucomannan on a Tumor Immune Microenvironment

Progression of tumor into malignant stage is closely associated with a compromised immune surveillance function, wherein tumor-associated macrophages (TAMs) are predominantly involved (Zhan et al., 2014). Actually, glucomannan from Porang was demonstrated to activate macrophages through Toll-like receptor 4 (TLR4), increasing the production of interleukin-6 (IL-6) and tumor necrosis factor α (TNFα) and promoting its phagocytic capability in primary macrophages (Gurusmatika et al., 2017) (**Figure 3**). On the other hand, ubiquitous surface expression of glucose and mannose receptors makes TAMs possible targets for polysaccharides with anti-tumor activity (Chorvatovicova et al., 1999; Dong et al., 2009; Zhan et al., 2014). Although no specific study indicates that the glucomannan binding to the sugar receptor has direct influence on macrophage, studies emphasize the intermediary role of glucomannan as a TAM-targeting molecule. Glucomannan isolated from *B. striata* (BSP) shows nucleic acid binding affinity after modification by *N*,*N*’-carbonyl di-imidazole (CDI)/ethylene diamine (Zhan et al., 2014). The anti-sense nucleotides such as oligo-deoxynucleotide and small interference RNA (siRNA) could be delivered by the modified glucomannan *via* the recognition of macrophage surface receptors, which mediates the manipulation of TAM-related gene expression level (Dong et al., 2009). A study shows that glucomannan helps to selectively deliver alendronate to tumor-resident macrophages, thus leading to the apoptosis and depletion of TAMs (Zhan et al., 2014) (**Figure 3**). Also, the bisphosphonate glucomannan conjugate, consisting of poly-ethylene glycol (PEG), poly(lactic-co-glycolic acid) (PLGA), and a peptide that can be cleaved by matrix metalloproteases (MMPs), could be efficiently released to tumor sites and reduce the viability of TAMs.

Moreover, glucomannan was demonstrated to enhance the immune function both *in vivo* and *in vitro* (Zhan et al., 2014). Interleukin-10 (IL-10) is known as an immunosuppressive cytokine that is enriched in tumors to counteract the killing activity of cytotoxic T lymphocytes (CTLs) (Salazar-Onfray, 1999). Glucomannan could re-energize the immune system to attack the tumor cells by decreasing IL-10 level and promoting the production of IFN-γ in tumor sites (Suzuki, 1983; Zhan et al., 2014). Similarly, the acidolysis-oxidized Konjac glucomannan up-regulates the expression of cytokines like TNFα, interleukin-1β (IL-1β), and IL-6, which collectively bolster the anti-tumor immune response (Vazquez-Velasco et al., 2015; Chen et al., 2017b) (**Figure 3**). However, it is important to note that glucomannan has also been suggested to possess an anti-inflammatory effect (Onishi et al., 2007; Wu et al., 2011a). Onishi N et al. indicated that pulverized konjac glucomannan suppressed the skin inflammatory immune response in NC/Nga mice evidenced by decrease of substance P, IL-10, IL-4, and TNFα (Onishi et al., 2007). Thus, whether the immune regulatory function of glucomannan is bidirectional or the immune motivating role is specific in local tumor environment remains to be determined by future studies.

## Discussion

As a straight-chain polymer with few branches, glucomannan was first appreciated for its role in gastroenterological disorders and metabolic diseases. Much similar to other polysaccharide extracts from traditional Chinese herbs, recently, the potential of glucomannan involved in anti-cancer therapy is being revealed. Accumulating evidence suggests that glucomannan exhibits broad but specific anti-tumor effect, when distinct cancer types are concerned (**Table 1**).

**Table 1:** The anti-tumor mechanism of glucomannan in different cancer types.

Source of GM	Cancer type	Model		Mechanism
*Amorphophallus konjac*	Breast cancer (Wu et al., 2018; Wu et al., 2019)	*In vitro*	MDA-MB-231	Induce cell cycle arrest
			MDA-MB-231BO	Inhibit migration and invasion
		*In vivo*	Mice	Regulation of the chromosomal and centrosomal instability
	Hepatoma (Ansil et al., 2013; Ansil et al., 2014c; Ansil et al., 2014b; Sawai et al., 2018)	*In vitro*	PLC/PRF/5	Anti-proliferation
			HepG2	Increase apoptosis
		*In vivo*	Rat	Anti-oxidative stress
				Decrease cell viability
				Promote the production of propionate
		*In vitro*	SGC-7901	Increase apoptosis
	Gastric cancer (Chen et al., 2017a)		AGS	Induce cell cycle arrest
		*In vivo*	Human	Promote autophagy
	Colon carcinoma (Wu and Chen, 2011b; Ansil et al., 2013; Ansil et al., 2013; Ansil et al., 2014a; Wu et al., 2014)	*In vitro*	HCT-15	Induce apoptosis
				Anti-proliferation
		*In vivo*	Rat	Anti-oxidative stress
				Reduce β-glucuronidase and mucinase activities
				Promote the growth of *bifidobacteria* and *lactobacilli* and production of SCFAs
	Lung cancer	*In vivo*	Mice	—
*Candida utilis*	Lung cancer (Kumano et al., 1985)	*In vivo*	Mice	Inhibit migration and invasion
	Leukemia (Miadoková et al., 2006)	*In vitro*	P388D1	Anti-oxidative stress
*Lentinus edodes*	Ehrlich ascites tumor	*In vivo*	Mice	Induce expression of interferons
	Sarcoma S180-bearing (Fujii et al., 1978)			
*Bletilla striata*	Sarcoma S180-bearing (Zhan et al., 2014)	*In vivo*	Mice	Targeted depletion of TAMs
*Aloe vera*	Colon carcinoma (Im et al., 2016; Quezada et al., 2017)	*In vitro*	HT29	Promote the growth of *bifidobacteria* and *lactobacilli* and production of SCFAs
		*In vivo*	Mice	Induce cell cycle arrest
	Leukemia (Sampedro et al., 2004)	*In vitro*	C1498	Anti-proliferation
	Ehrlich ascites tumor	*In vivo*	Mice	Activate macrophages
	Sarcoma 180-bearing (Im et al., 2005; Liu et al., 2006)			

SCFAs, short-chain fatty acids; TAMs, tumor-associated macrophages.

Mechanistically, glucomannan has direct impact on tumor cell survival and metastasis by blocking the PI3K/AKT signaling pathway (Frisch and Screaton, 2001; Guanen et al., 2018). After glucomannan treatment, cells show increased apoptosis along with decreased proliferation capacity. Meanwhile, the expression of chemokine receptors (CCR7 and CXCR4) is reduced, which abrogates the migratory ability of the tumor cells (Wu et al., 2018). Uncontrolled cellular stress is pivotal in the early stage of tumorigenesis. Notably, hyperactive tumor metabolism leads to elevated ROS level and subsequent oxidative stress, which would cause genotoxicity and accumulation of mutations required for tumor development (Wu et al., 2014). Autophagy is a stress response characterized by degradation of self-components and the formation of autophagosomes. During early-stage tumor development, autophagy exerts a tumor-suppressive function through the clearance of stress-induced organelle damage. Glucomannan displays a protective effect by promoting the expression of genes associated with ROS scavenging and autophagy induction (Chen et al., 2017a). Though the underlying mechanism remains obscure, it is plausible to reason that the PI3K/AKT signaling pathway may also be critically involved in such stress responses. Studies show that inhibition of the PI3K/AKT pathway limits the glycolytic process and deprives tumor cells of energy supply (Chen et al., 2016; Cretella et al., 2018), which could explain the reduction of ROS content (Robey and Hay, 2009; Zhao et al., 2017) and the elevation of cell autophagy level (Jin and White, 2007; Cheong, 2015; Liu et al., 2017). Altogether, glucomannan ameliorates cellular stress and prevents the tumorigenesis process.

Other than the direct interference of tumor cell biology, glucomannan also functions in an indirect manner. Glucomannan administration reduces carcinogen production in gut lumen and promotes probiotics and SCFA generation, which suppresses GI inflammation-related carcinogenesis (Wu et al., 2011a). On the other hand, due to its excellent bio-compatibility, glucomannan can work as a targeted delivery platform to enhance the efficiency of traditional drugs like curcumin (Luan et al., 2017), teniposide (Miadoková et al., 2006), and 5-FU (Wang et al., 2014). Anergic immune microenvironment is an important culprit in the process of tumor growth and evasion; thus, current therapies like checkpoint inhibitors are designed to motivate and re-energize the attacking ability of effector immune cells. Strikingly, glucomannan could alter the cytokine profile in the tumor vicinity and thus in support of CTL-mediated cytotoxic effect. TAMs, tumor resident macrophages that contribute to the induction of a tolerant immune milieu, express receptors for various polysaccharide molecules. Glucomannan could increase macrophage phagocytic activity and cytokine secretion, such as TNFα and IL-6, *via* binding to TLR4 (Gurusmatika et al., 2017). It also facilitates the function of other compounds like siRNA (Dong et al., 2009) and alendronate (Zhan et al., 2014) to induce TAM apoptosis. All in all, further studies are needed to understand the details of how glucomannan impacts the immune system.

Cancer, the primary killer in modern society, is a complicated and systematic disease. The ultimate goal in cancer therapy is to completely eradicate tumor cells and reach the criteria of clinical cure without disease recurrence. It is a challenging task, considering the presence of cancer stem cells, intra-tumoral heterogeneity, and the formidable adaptability of tumor cells. While in pursuit of such purpose, it is also important to render patients live in “harmony” with their tumors, which means to maintain the high quality of life amid the effort to stop tumor from deterioration. According to a report published in *JAMA Oncology* in 2019, 1/3 of cancer patients would seek for help from alternative medicines (Sanford et al., 2019). Nature holds the key to cancer management, and ethnopharmacological herbs may be one of the answers that nature provides. Therefore, the identification, synthesization, and modification of additional bioactive anti-tumor compounds like glucomannan from natural ethnopharmacological herbs would greatly benefit the process of anti-tumor therapy and contribute to the improvement of human health.

## Author Contributions

J-YL, FS, and H-FZ proposed and wrote the manuscript. JY and CH collected and analyzed the information. HF supervised the conception and writing of the article.

## Funding

The research was supported by the NSFC grant number 81774093.

## Conflict of Interest Statement

The authors declare that the research was conducted in the absence of any commercial or financial relationships that could be construed as a potential conflict of interest.
